# Appraisal of Antioxidant and Anti-Inflammatory Activities of Various Extracts from the Fruiting Bodies of *Pleurotus florida*

**DOI:** 10.3390/molecules19033310

**Published:** 2014-03-18

**Authors:** Kyung Hoan Im, Trung Kien Nguyen, Do Bin Shin, Kyung Rim Lee, Tae Soo Lee

**Affiliations:** Division of Life Sciences, Incheon National University, (Songdo-dong) 119 Academy-ro, Yeonsu-gu, Incheon 406-772, Korea

**Keywords:** antioxidant, phenolic compounds, *Pleurotus florida*, anti-inflammation activity

## Abstract

*Pleurotus florida* has been widely used for nutritional and medicinal purposes. The present study was conducted to evaluate the antioxidant and anti-inflammatory effects of the fruiting bodies of *P*. *florida* extracted with acetone, methanol, and hot water. The antioxidant activities of the acetone and methanol extracts of *P*. *florida* showed stronger inhibition of β-carotene-linoleic acid compared to that of the hot water extract. The acetone extract (8 mg/mL) showed a high reducing power of 1.86. The acetone and methanol extracts showed more effective DPPH radical scavenging activities than the hot water extract. The chelating effect of the extracts at lower concentrations was significantly effective compared to that of the positive control. Thirteen phenolic compounds were detected from acetonitrile and hydrochloric acid solvent extracts. Nitric oxide (NO) production and inducible nitric oxide synthase (iNOS) expression in lipolysaccahride (LPS) stimulated RAW 264.7 cells, a murine macrophage cell line, were inhibited significantly by the mushroom extracts in a concentration dependent manner. The anti-inflammatory activity on carrageenan-induced edema in the rat hind-paw reduced significantly by the mushroom extracts. Therefore, we have demonstrated that *P*. *florida* fruiting bodies possess antioxidant and anti-inflammatory activites related to their inhibitory activities on NO production, iNOS protein expression, and carrageenan-induced paw edema in rats. The results suggest that the fruiting bodies of *P*. *florida* are a good source of natural antioxidant and anti-inflammatory agents.

## 1. Introduction

*Pleurotus florida* is an edible mushroom that belongs to the family Pleurotaceae, order Agaricales, Basidiomycota. This mushroom produces various biologically active molecules and novel enzymes [1]. Oxidation is essential for producing energy to fuel biological processes. However, free radicals and other reactive oxygen species (ROS) that are continuously produced *in vivo* can cause cell death and tissue damage. Oxidative damage caused by free radicals may be related to aging and diseases such as atherosclerosis, diabetes, cancer, and cirrhosis [2]. Almost all organisms are well protected against free radical damage by enzymes, such as superoxide dismutase and catalase, or compounds such as ascorbic acid, α-tocopherols, and glutathione. Although almost all organisms possess antioxidant defense and repair systems that have evolved to protect them against oxidative damage, these systems are insufficient to prevent the damage entirely [3]. Therefore, antioxidant supplements or foods containing antioxidants may be useful to help the human body reduce oxidative damage. Crude flavonoid and polyphenolic extracts possess inhibitory activity on oxidative enzymes.

Inflammation is a physiological immune response of the host against internal and external stimuli such as infection, tissue injury, or noxious stimulants; however, the over-production of inflammatory products can lead to a series of vascular and cellular reactions [4]. Inflammatory markers such as ROS, reactive nitrogen species, tumor necrosis factor-α, interleukin (IL)-1, IL-6, and cyclooxygenase (COX) are produced under stress [5]. Chronic accumulation of inflammatory products can mediate a wide variety of diseases such as atherosclerosis, rheumatoid arthritis, diabetes, multiple sclerosis, and Alzheimer’s disease [6]. Inducible nitric oxide synthase (iNOS) catalyzes the formation of nitric oxide (NO) from l-arginine [7] and is responsible for increased production of NO during inflammation. NO is a free radical, produced by activated macrophages that mediate many physiological and pathophysiological processes including inflammation [8]. Thus, inhibiting iNOS activity and NO production in macrophage cells are very important during the anti-inflammatory process.

Despite the clinical and therapeutic importance of *P. florida*, a few studies on its physiologically beneficial components have been conducted, and the antioxidant and anti-inflammatory properties of this mushroom are not available in the literature. Accordingly, our objective was to evaluate and compare the antioxidant and anti-inflammatory activities of acetone, methanol, and hot water extracts of the fruiting bodies of *P. florida*. The phenolic compound profiles of the mushroom extracts were also determined.

## 2. Results and Discussion

### 2.1. Evaluation of Antioxidant Activity

#### 2.1.1. Antioxidant Activity on β-Carotene-linoleic Acid

The antioxidant activities of the acetone, methanol, and hot water extracts from the fruiting bodies of *P*. *florida* on β-carotene-linoleic acid increased gradually with increasing concentration. At 0.5–20.0 mg/mL, the antioxidant activities of acetone, methanol, and hot water extracts of *P*. *florida* were 36.47%–91.62%, 31.56%–91.41%, and 36.74%–90.42%, respectively (Table 1). These results indicate lower antioxidant activities of *P. florida* than those of the synthetic antioxidants BHT and TOC at 0.5 mg/mL. The acetone and methanol extracts showed good activity, whereas the hot water extract showed moderate activity at the concentrations tested. The antioxidant activity of carotenoids is based on the formation of adducts of the carotenoids with the free radicals from linoleic acid. The linoleic acid free radical attacks the highly unsaturated β-carotene. The presence of the carotenoid results not only in a decrease of free radicals, but a reduction of Fe^3+^ to Fe^2+^ by carotenoids. It is probable that the antioxidative components in the mushroom extracts can reduce the extent of β-carotene destruction by neutralizing the linoleate free radical and other free radicals formed in the system [9]. Barros *et al*. [10] reported that the antioxidant activities of various extracts from *Leucopaxillus giganteus*, *Sarcodon imbricatus,* and *Agaricus arvensis* increased with increasing extract concentration. Their antioxidant activities were 61.4%, 54.3% and 46.7% at 5 mg/mL, whereas the antioxidant activity of the TBHQ (tertiary butylhydroquinone) standard reached 82.2% at 2 mg/mL concentration. It seems that the antioxidant activity of *P. florida* fruiting bodies was more effective than those mentioned above.

**Table 1:** Antioxidant activity against β-carotene-linoleic acid of different concentrations of various extracts from the fruiting bodies of *Pleurotus florida*.

Solvent and Control	Sample Concentration (mg/mL)			
	0.5	2.0	8.0	20.0
Acetone	36.47 ± 0.11	77.05 ± 0.72	80.92 ± 0.54	91.62 ± 0.04
Methanol	31.56 ± 0.05	71.11 ± 0.14	87.99 ± 0.16	91.41 ± 0.08
Hot water	36.74 ± 0.14	69.04 ± 0.12	84.80 ± 0.14	90.42 ± 0.06
BHT	95.21 ± 0.17	-	-	-
TOC	96.02 ± 0.18	-	-	-

Values expressed as means ± SD (*n* = 3); -, not analyzed; BHT, butylated hydroxytoluene; TOC, α-tocopherol.

#### 2.1.2. Reducing Power

The reducing power of the *P. florida* acetone, methanol, and hot water extracts increased with increasing concentration. The strongest reducing power inhibition (1.95) was observed for 8 mg/mL acetone extract, and the lowest reducing power inhibition (1.88) was exhibited by the hot water extract. The synthetic antioxidants BHT and TOC exhibited higher reducing power values of 3.21 and 2.16, respectively, at 1.0 mg/mL (Table 2). Menaga *et al*., [11] reported that methanol extract of *P. florida* fruiting bodies showed reducing power of 0.911 at 0.5 mg/mL, which was significantly higher than reducing power of our methanol extract of 0.87 at 1 mg/mL. The reducing power of *P. florida* cultivated in Bangladesh was 68.69 μg of ascorbic acid equivalent of mg of extract, which was a little lower than that of our *P. florida* reducing power [12]. The reducing power of a hot water extracts of *Hypsizygus marmoreus* was 0.99 at 5 mg/mL, whereas those of *Agaricus bisporus*, *Pleurotus eryngii*, *Pleurotus ferulae* and *Pleurotus ostreatus* showed reducing powers of 0.76, 0.75, 0.70, and 0.61 at 20 mg/mL, respectively [13]. 

**Table 2:** Reducing power of different concentrations of various extracts from the fruiting bodies of *Pleurotus florida*.

Solvent and Control	Sample Concentration (mg/mL)			
	1.0	2.0	4.0	8.0
Acetone	0.76 ± 0.05	1.32 ± 0.06	1.83 ± 0.03	1.95 ± 0.07
Methanol	0.87 ± 0.22	1.46 ± 0.21	1.82 ± 0.22	1.94 ± 0.19
Hot water	0.54 ± 0.18	0.73 ± 0.17	1.09 ± 0.16	1.88 ± 0.28
BHT	3.212 ± 0.49	-	-	-
TOC	2.162 ± 0.32	-	-	-

Values expressed as means ± SD (*n* = 3); -, not analyzed; BHT, butylated hydroxytoluene; TOC, α-tocopherol.

Our results indicate that the reducing power of *P. florida* was significantly more effective than those of the above mentioned mushrooms. The reducing power of *P. florida* cultivated in India was a little higher than that of cultivated in Bangladesh. Reducing power properties are generally associated with the presence of reductones, which exert their antioxidant action by breaking the free radical chain and donating a hydrogen atom [10,14].

#### 2.1.3. DPPH Scavenging Effect

**Figure 1:**
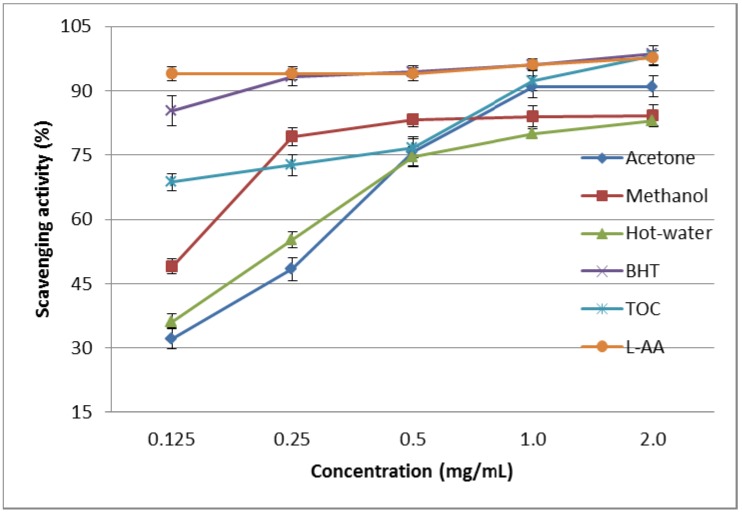
Scavenging activity of various extracts from the fruiting bodies of *Pleurotus florida* against 1,1-diphenyl-2-picrylhydrazyl. Values expressed as means ± SD (*n* = 3). BHT, butylated hydroxytoluene; TOC, α-tocopherol; L-AA, l-ascorbic acid.

The scavenging effects of the acetone, methanol, and hot water extracts from the fruiting bodies of *P. florida* on DPPH radicals increased with increasing concentration. At 0.125–2.0 mg/mL, the scavenging abilities of the acetone, methanol, and hot water extracts on the DPPH radical ranged from 32.62%–90.52%, 48.33%–84.13%, and 35.35%–81.81%, respectively (Figure 1). These results indicate that the acetone extract possessed good activity, whereas the methanol and hot water extracts showed moderate and poor activity, respectively, at the concentrations tested. However, at 0.125–2.0 mg/mL, BHT, TOC, and l-ascorbic acid showed excellent scavenging abilities of 85.25%–98.74%, 67.37%–97.78%, and 96.74%–98.23%, respectively.

DPPH scavenging activity of methanol extract on *P. florida* harvested in India was 78% at the concentration of 0.1 mg/mL, whereas the DPPH activity of methanol extract of *P. florida* was 47.5% at 0.125 mg/mL. Methanol extracts of *H. marmoreus*, *A. bisporus,* and *Pleurotus citrinopileatus* fruiting bodies scavenged DPPH radicals by 46.6%–68.4% at 5 mg/mL [11]. The scavenging activities of 20% mg/mL cold and hot water extracts of fruiting bodies, mycelia, and filtrate were 20.7%–52.3%, 37.6%–48.3%, and 19.6%–23.3%, respectively. It seemed that the scavenging activity of *P. florida* fruiting bodies was more effective than those mentioned above, except for *P. florida* cultivated in India. Various extracts might react with free radicals, particularly peroxy radicals, which are major propagators of fat autoxidation, thereby terminating the chain reaction [15]. Antioxidant activity of natural antioxidants is involved in terminating the free radical reaction [14]. Furthermore, Herraiz *et al*. [16] found that the essential amino acid l-tryptophan reacts with phenolic aldehydes in food to form phenolic tetrahydro-β-carboline alkaloids that scavenge 2,2-azinobis(3-ethylbenzothiazoline)-6-sulfonic acid effectively. Therefore, the presence of l-tryptophan in various extracts might account for their scavenging activity on DPPH radicals. However, the better activity of the acetone extract may have been due to more hydrogen-donating components contained within the extract.

#### 2.1.4. Chelating Effects on Ferrous Ions

**Figure 2:**
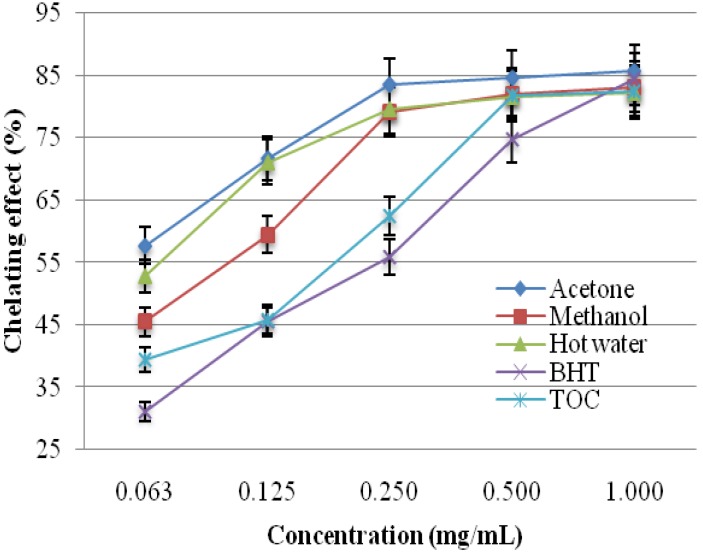
Chelating effect of various extracts from the fruiting bodies of *Pleurotus florida*. Values expressed as means ± SD (*n* = 3). BHT, butylated hydroxytoluene; TOC, α-tocopherol.

The chelating activity of the acetone, methanol, and hot water extracts from the fruiting bodies of *P. florida* toward ferrous ions was investigated at five different concentrations (0.063, 0.125, 0.250, 0.500, and 1.000 mg/mL). BHT and TOC were used as reference standards for ferrous ions. As shown in Figure 2, the chelating capacity of the extracts increased with increasing extract concentration. The strongest chelating effect (85.66%) was obtained from the acetone extract at 1.0 mg/mL. At this concentration, the lowest chelating effect was exhibited by the hot water extract (82.29%). The ferrous ion chelating activities of methanol extract *P. florida* was 64% at the concentration of 0.5 mg/mL [11].

Hot water extracts of 20 mg/mL from *Ganoderma tsugae* and *Agrocybe cylindracea* chelated ferrous ions by 42.6 and 45.8%, respectively [17,18]. The chelating abilities of *H. marmoreus* and *P. citrinopileatus* at 1–5 mg/mL were 75.6%–92.6% [19]. It seems therefore that the chelating ability of *P. florida* on ferrous ions was similar to that of *H. marmoreus* and *P. citrinopileatus* but more effective than those of *G. tsugae* and *A. cylindracea* and *P. florida* grown in India. Chelating agents may serve as secondary antioxidants because they reduce the redox potential thereby stabilizing the oxidized form of metal ions. As ferrous ions are the most effective pro-oxidants in food systems [20], the high ferrous-ion chelating abilities of the various extracts from the fruiting bodies of *P. florida* would be beneficial.

### 2.2. HPLC Analysis of Phenolic Compounds

**Figure 3:**
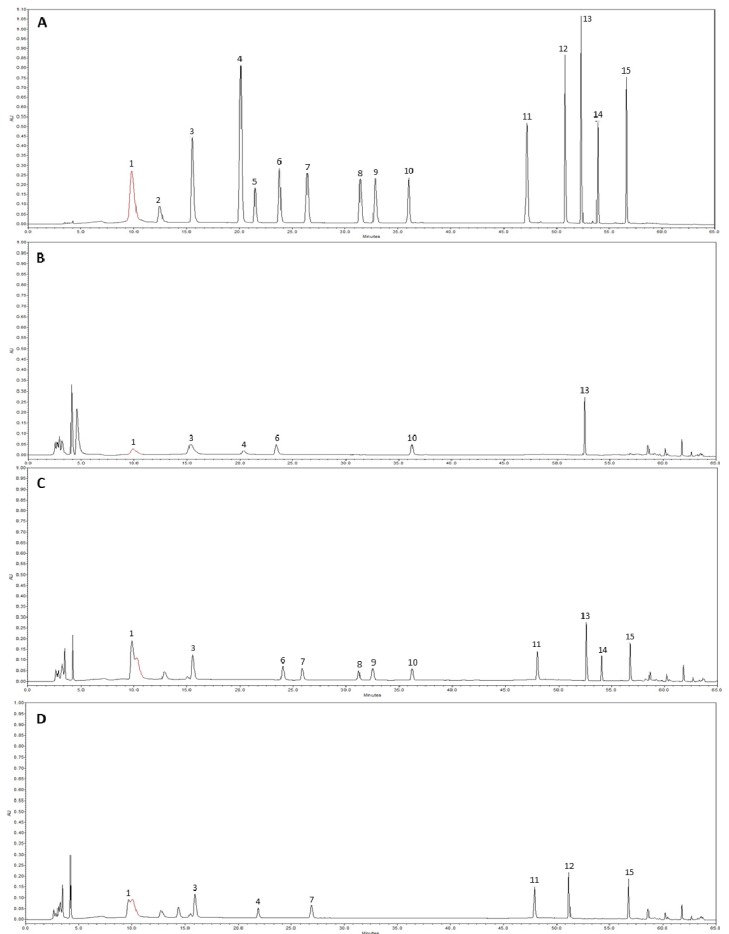
High performance liquid chromatography of phenolic compounds. (**A**) standard mixture of 15 phenolic compounds; (**B**) methanol extract; (**C**) acetone extract; (**D**) hot-water extract. 1, gallic acid; 2, pyrogallol; 3, homogentisic acid; 4, protocatechuic acid; 5, (+)-catechin; 6, chlorogenic acid; 7, caffeic acid; 8, vanillin; 9, ferulic acid; 10, naringin; 11, resveratrol; 12, naringenin; 13, hesperetin; 14 fomononetin; 15, biochanin-A.

Thirteen of the 15 phenolic compounds monitored were detected, and only pyrogallol and (+)-catechin were not detected in the studied mushroom extracts (Figure 3). The numbers of phenolic compounds detected in methanol, acetone and hot water extracts were 6, 11 and 7, respectively. Overall, the phenolic compounds concentration was greater in the acetone extract compared with the methanol and hot water extracts. The total average concentration of phenolic compound in the methanol, acetone and hot water extracts were 77.55, 135.08 and 62.53 µg/g, respectively. The thirteen phenolic compounds detected from the three mushroom extracts were gallic acid, homogentisic acid, protocatechuic acid, chlorogenic acid, caffeic acid, vanillin, ferulic acid, naringin, resveratrol, naringenin, hesperetin, formononetin, and biochanin-A (Figure 3B–D). The highest and lowest concentrations of phenolic compounds were gallic acid in the acetone extract (32.60 µg/g) and protocatechuic acid in the hot water extract (1.54 µg/g).

These findings were comparable to those of previous studies on edible mushrooms [21] in which the total concentration of phenolic compounds was 174 µg/g. Mushroom species also contain different numbers of phenolic compounds ranging from 3 to 15, with gallic acid being the most common phenolic compound in mushrooms. Thus, phenolic compound content could be used as an important indicator of antioxidant capacity. Several reports have convincingly shown a close relationship between antioxidant activity and phenolic content [22,23,24]. Mushroom extracts have high levels of phenolic compounds, which are composed of one or more aromatic rings bearing one or more hydroxyl groups that exhibit extensive free radical-scavenging activities as hydrogen donors or electron-donating agents, as well as metal ion-chelating properties. The greater numbers of hydroxyl groups in the phenolic compounds could result in higher antioxidant activity [25,26].

### 2.3. Evaluation of Anti-Inflammatory Activity

#### 2.3.1. NO Production

**Figure 4:**
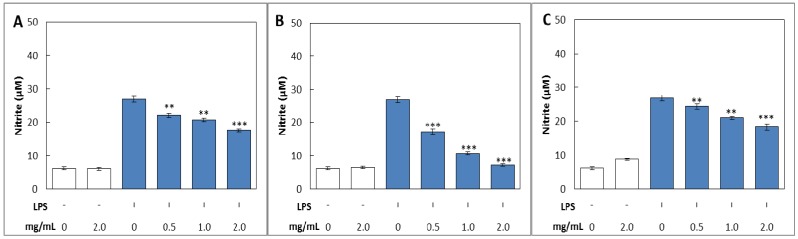
Inhibitory effects of various extracts from fruiting bodies of *Pleurotus florida* on LPS-induced nitrite production in RAW 264.7 cells. (**A**); methanol extract, (**B**); acetone extract and (**C**); hot-water extract. Accumulated nitrite in the culture medium was determined by the Griess reagent. The values are expressed as ± SD (*n* = 3). *** *p* ≤ 0.001; ** *p* ≤ 0.01 *vs*. group treated with LPS alone.

The accumulated concentration of NO in the culture medium was measured by the Griess reaction [27]. After treatment with LPS on RAW 264.7 cells for 24 h, concentration of NO markedly increased about 4.40–fold (6.10–26.87 µM), whereas the NO production of cells treated with various concentrations of methanol, acetone and hot water extracts of fruiting bodies of *P. florida* were significantly inhibited at the concentrations from 0.5 to 2 mg/mL concentration in a dose dependent manner (Figure 4). The NO production on RAW 264.7 cells treated only with acetone extract of 2 mg/mL was 6.44 µM, which was comparable with 6.10 µM of LPS non-treated control group, whereas NO production in LPS stimulated RAW 264.7 cells treated with acetone extract of 2 mg/mL was 7.15, which was 1.17–fold higher than that of the control group (Figure 4B). The LPS-induced RAW 264.7 cells treated with methanol extract of 2 mg/mL significantly decreased the production of NO by 34.80% compared with LPS only treated group (Figure 4C). The LPS-induced RAW 264.7 cells treated with hot water extract of 2 mg/mL concentration significantly decreased the production of NO by 31.97% compared with LPS only treated group (Figure 4C). From the results, it is concluded that inhibitory activity of NO production in acetone extract on LPS-induced RAW 264.7 cells was better than those of methanol and hot water extract tested.

No cytotoxicity was observed at the concentrations tested as determined by an MTT test (data not shown). From this result, it is expected that the inhibition of NO production by mushroom extracts in RAW 264.7 cells would be caused by decreased iNOS protein expression. Park and Won [28] reported that ethanol extracts of cultured mycelia and fruiting body of *Cordyceps militaris* suppressed production of NO and iNOS protein in LPS-stimulated RAW 264.7 macrophages in a concentration dependent manner. Furthermore, Kim *et al*. [29] found that an *n*-butanol sub-fraction of *P*. *linteus* fruiting bodies also inhibited NO and iNOS protein in LPS-activated RAW 264.7 cells. It seemed that inhibitory effect of NO production by extracts of *P*. *florida* fruiting bodies in LPS-induced RAW 264.7 cells was similar to that of *C*. *militaris* and *P*. *linteus*. 

#### 2.3.2. Western Blot Analysis

**Figure 5:**
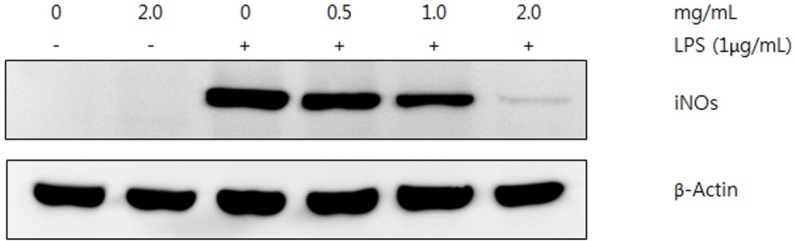
Inhibitory effect of acetone extract from fruiting bodies of *Pleurotus florida* on LPS-induced expression of iNOS protein. RAW 264.7 cells were incubated with LPS (1 µg) in the presence or absence of indicated concentration of mushroom. β-Actin was used as an internal control.

Various extracts from the fruiting bodies of *P*. *florida* showed reduced production of NO in LPS-induced RAW 264.7 macrophages in a dose dependent manner. With the assumption that the suppressed effect of NO production in RAW 264.7 cells would be caused by inhibition of iNOS protein expression, we conducted western blot analysis to evaluated iNOS protein production. Since acetone extract of the mushroom contained more phenolic compounds compared with the methanol and hot water extracts, and showed the better antioxidant and anti-inflammatory compared to other two extracts, western blot analysis was conducted using the acetone extract. The iNOS protein in RAW 264.7 cells activated by LPS was markedly decreased by treatment with the mushroom extract in a concentration dependent manner, whereas no change was found on β-actin, an internal control, indicating that the specific inhibition on the iNOS protein expression by the acetone extract of the mushroom (Figure 5). The results suggested that mushroom extract was responsible for reduced production of NO and iNOS protein. Therefore, the inhibitory effect of NO production was directly related to down-regulation of iNOS by the mushroom extracts in LPS-activated RAW 264.7 macrophages.

#### 2.3.3. Carrageenan-Induced Rat Paw Edema

**Figure 6:**
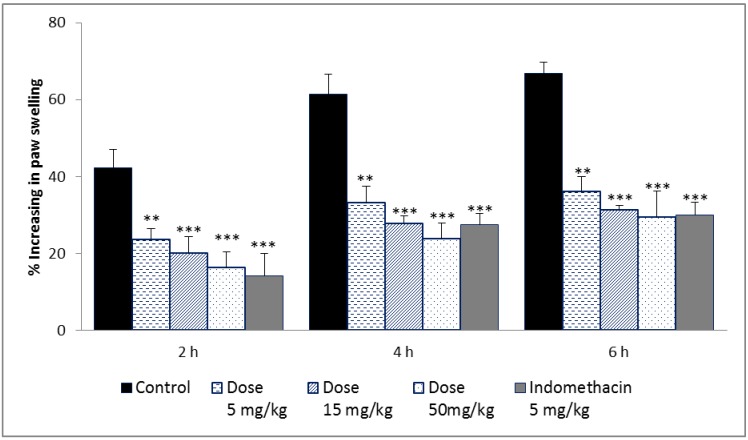
Effect of acetone extract from fruiting bodies of *Pleurotus florida* on carrageenan-induced edema in rat hind-paw. Indomethacin wad used as positive control. The values are expressed as ±SD (*n* = 5). *** *p* ≤ 0.001; ** *p* ≤ 0.01 *vs*. control group.

The carrageenan-induced rat paw edema model has been used to evaluate anti-inflammatory activity *in vivo*. As shown in Figure 6, intraperitoneal administration of indomethacin, the positive control, resulted in a significant reduction in rat paw edema at 6 h (55.08%), whereas the 5, 15, 50 mg/kg acetone extract reduced paw edema by 45.81%, 53.01% and 55.97%, respectively. The anti-inflammatory effect of acetone extract at doses of 5–50 mg/kg was statistically significant for reducing paw edema of rats at 2 and 4 h after induction of edema. The inhibitory effect of the acetone extract appeared to be comparable to that of indomethacin. Carrageenan-induced edema develops through mediators in three phases. The early phase is caused by histamine release, the second phase is mediated by kinin, and the late phase is caused by prostaglandins [30]. Most anti-inflammatory drugs are effective for the last phase of edema formation [31]. Therefore, the potent antioxidant and anti-inflammatory activities found in *P. florida* fruiting bodies can be used for treatment of oxidative stress-induced inflammatory disorders.

## 3. Experimental

### 3.1. Chemicals and Reagents

β-Carotene, linoleic acid, chloroform, polyoxyethylene sorbitan monopalmitate (Tween 40), BHT, TOC, DPPH, l-ascorbic acid, potassium ferricyanide, trichloroacetic acid, ferrous chloride, ferric chloride, ferrozine, Folin–Ciocalteu reagent, gallic acid, methanol, 3,4-dihydroxy-l-phenylalanine, dimethyl sulfoxide (DMSO), tris base, glacial acetic acid, trichloroacetic acid, carrageenan, and *Escherichia coli* lipolysaccharires (LPS) were obtained from Sigma-Aldrich (St. Louis, MO, USA), as were the phenolic compound standards. All chemicals and solvents used were high perfromace liquid chromatography (HPLC) or analytical grade.

### 3.2. Animals

Five week old inbred male Sprauge Dawley rats (140–160 g) were purchased from Central Lab. Animal Inc., Seoul, Korea. All rats were acclimated to the animal house for a period of 1 week. The rats were housed in an animal room at 23 ± 2 °C under 12 h dark-light cycles (17:00–05:00) and a relative humidity of 50%–60%. During the experimental period, standard basal diet and water were given *ad libitum* to rats. The experimental protocol was approved by the Animal Ethics Committee of Incheon National University.

### 3.3. Mushrooms and Extraction

Fresh and mature fruiting bodies of *P. florida* were hot air dried at 40 °C for 48 h and finely pulverized. Five g of powdered sample was extracted with 60% acetone and 80% methanol (100 mL each) with stirring at 150 rpm for 24 h at 25 °C to obtain the acetone and methanol extracts. The mixture was filtered through two layers of Whatman No. 1 filter paper (Whatman, Maidstone, UK). The same quantity of sample was boiled at 100 °C for 3 h with deionized distilled water (100 mL) to obtain the hot water extract. The mixture was cooled to room temperature and filtered through Whatman No. 1 filter paper. The residues were then extracted with two additional 100 mL aliquots of acetone, methanol, and deionized water, as described above. The combined extracts were evaporated with a rotary evaporator at 40 °C, and the remaining solvent was removed with a freeze-drier. Yields of the acetone, methanol, and hot water extracts of *P. florida* were 22.24%, 24.18%, and 18.25% (w/w), respectively.

### 3.4. Evaluation of Anti-Oxidant Activity

#### 3.4.1. Antioxidant Activity due to β-Carotene-linoleic Acid

Antioxidant activity was determined by measuring inhibition of volatile organic compounds and the conjugated diene hydroperoxides arising from linoleic acid oxidation [32]. A stock solution of a β-carotene-linoleic acid mixture was prepared as follows: β-carotene (0.5 mg) was dissolved in chloroform (1 mL), and linoleic acid (25 µL) and Tween 40 (200 mg) were added. The chloroform was removed completely using a vacuum evaporator. Then, oxygenated distilled water (100 mL) was added with vigorous shaking; a portion of this reaction mixture (2.5 mL) was dispensed into test tubes, various concentrations (0.5–20.0 mg/mL, 0.5 mL) of the extracts in methanol was added, and the reaction mixture was incubated for up to 2 h at 50 °C. The same procedure was repeated with the positive controls BHT and TOC, and a blank. After the incubation, the absorbance of the mixtures was measured at 490 nm using a spectrophotometer (Optizen pop, Daejeon, Korea). The absorbance was measured until the β-carotene color disappeared. The β-carotene bleaching rate (R) was calculated according to Equation (1):

R = ln(a/b)/t
(1)

where, ln is natural log, a is absorbance at time t (0), and b is absorbance at time t (120 min). Antioxidant activity (AA) was calculated as percent inhibition relative to the control using Equation (2):

AA = [(R_control_ − R_sample_)/R_control_] × 100 (2)

Antioxidant activities of the extracts were compared with those of BHT and TOC at 0.5 mg/mL and a blank consisting of 0.5 mL methanol.

#### 3.4.2. Reducing Power

Reducing power was determined according to the method of Gülçin *et al*. [33]. Each extract (1–8 mg/mL) in methanol (2.5 mL) was mixed with 200 mM sodium phosphate buffer (pH 6.6, 2.5 mL) and 1% potassium ferricyanide (2.5 mL), and the mixture was incubated at 50 °C for 20 min. Then, 10% trichloroacetic acid (2.5 mL) was added, and the mixture was centrifuged at 200 *×g* (6K 15; Sigma, Munich, Germany) for 10 min. The upper layer (2.5 mL) was mixed with deionized water (2.5 mL) and 0.1% ferric chloride (0.5 mL). Finally, the absorbance was measured at 700 nm against a blank. BHT and TOC were used as positive controls.

#### 3.4.3. Scavenging Effect on DPPH Radicals

The hydrogen atoms or electron donation ability of the extracts and some pure compounds were measured as bleaching of the purple colored DPPH methanol solution [34]. Four mL of various concentrations (0.125–2.0 mg/mL) of the extracts in methanol was added to DPPH radical solution in methanol (1 mL, final DPPH concentration, 0.2 mM). The mixture was shaken vigorously and allowed to stand for 30 min, and the absorbance of the solution was measured at 517 nm using a spectrophotometer. Inhibition of the DPPH free radical in percent (I%) was calculated as:

I% = [(A_control_ − A_sample_)/A_control_] × 100

where A_control_ is the absorbance of the control reaction (containing all reagents except the test compound), and A_sample_ is the absorbance of the test compound. BHT, TOC, and l-ascorbic acid were used as positive controls.

#### 3.4.4. Chelating Effects on Ferrous Ions

The chelating effect was determined according to the method of Dinis *et al*. [35]. Briefly, various concentrations (0.063–1.0 mg/mL, 2 mL) of the extracts in methanol was added to a solution of 2 mM FeCl_2_ (0.05 mL). The reaction was initiated by adding 5 mM ferrozine (0.2 mL). Total volume was adjusted to 5 mL with methanol, and the mixture was shaken vigorously and left at room temperature for 10 min. The absorbance of the solution was measured spectrophotometrically at 562 nm. The inhibition percentage of the ferrozine-Fe^2+^ complex formation was calculated using the following formula:

Metal chelating effect (%) = [(A_control_ − A_sample_)/A_control_] × 100

where A_control_ is the absorbance of the control (control contained FeCl_2_ and ferrozine; complex formation molecules), and A_sample_ is the absorbance of the test compound. BHT and TOC were used as positive controls.

### 3.5. HPLC Analysis of Phenolic Compounds

The phenolic compounds were analyzed using an Alliance^®^ HPLC system 2695 (Waters, Milford, MA, USA) equipped with a quaternary solvent pump and an automatic injector. Data acquisition and processing were carried out using the Waters Empower™ 2 software.

Fifteen standard phenolic compounds, including gallic acid, pyrogallol, homogentisic acid, protocatechuic acid, (+)-catechin, chlorogenic acid, caffeic acid, vanillin, ferulic acid, naringin, resveratrol, naringenin, hesperetin, fomononetin, and biochanin-A were used to prepare calibration curves. The standard stock solutions (25, 50, 75 and 100 ppm) were prepared in methanol. Sample compounds were identified based on retention times of authentic standards and were quantified by comparing their peak areas with those of the standard curves. 

An aliquot of 2 mg of each methanol, acetone and hot water extract was mixed with 2 mL of methanol and filtered through a 0.45 µm nylon membrane filter (Titan, Rockwood, TN, USA). The injection volume was 10 µL. Separation was achieved on a XSELECT CSH™ reverse phase C-18 column (150 mm × 4.6 mm × 3.5 µm) (Waters, Wexford, Ireland) maintained at 40 °C. The mobile phase was consisted of a gradient mixture of a solvent A (0.85% phosphoric acid solution) and solvent B (acetonitrile), with a flow rate of 0.5 mL/min. The gradient was started with 100% of solvent A and adjusted for 93% of solvent A and 7% of solvent B in 5 min; 91% of solvent A and 9% solvent B in 10 min; 85% of solvent A and 15% of solvent B in 15 min; 78% of solvent A and 22% of solvent B in 30 min; 75% of solvent A and 25% of solvent B in 40 min; 62% of solvent A and 38% of solvent B in 45 min; and 100% of solvent B in 60 min. Run time was 65 min. Detection of phenolic compounds was performed with Waters 2988 photodiode array detector at 210 nm as the preferred wavelength.

### 3.6. Evaluation of Anti-Inflammatory Activity

#### 3.6.1. NO Assay

RAW 264.7 cells were seeded onto 96-well plates with 5 × 10^5^ cells/well and allowed to overnight. Then, medium was removed and replaced with 0.2 mL of fresh medium and incubated for 1 h. Then, LPS (1 µg/mL) was supplemented to the medium and incubated in the presence or absence of the mushroom extracts for 24 h. The supernatant of culture medium was collected and 50 µL were used for NO determination. The nitrite (NO) accumulated in culture medium was measured Griess reagent. Briefly, 50 µL of cell culture medium were mixed with an equal volume of Griess reagent (equal volumes of 1 % (*w/v*) sulfanilamide in 5% (*v/v*) phosphoric acid and 0.1% (*w/v*) naphthylethylene-diamide–HCl), incubated at room temperature for 10 min, and then the absorbance was measured at 540 nm, using a microplate reader (SpectraMax 340PC, Sunnyvale, CA, USA). The amount of nitrite present in the samples was calculated by means of a standard curve was obtained using known concentrations of sodium nitrite.

#### 3.6.2. Western Blot Analysis

RAW 264.7 cells were incubated with LPS (1 µg/mL) in the presence or absence of the extracts for 24 h and then washed twice with ice-cold phosphate buffered saline (PBS, pH 8.0). The cells were lysed in a buffer containing 20 mM Tris-HCl (pH 7.5), 1% Triton X-100, 137 mM NaCl, 2 mM EDTA, 1 mM sodium orthovanadate, 2 mM sodium pyrophosphate, 1 mM phenylmethylsulfonyl fluoride, and 1 µg/mL leupeptin. Cell lysates were centrifuged at 10,000 *×g* for 10 min to remove cell debris. The supernatant was subjected to sodium dodecyl sulfate-polyacrylamide gel electophoresis on 8% polyacrylamide gels and transferred to a polyvinylidene difluride membrane in 25 mM Tris, 20% methanol and 192 mM glycine. The membranes were blocked with 5% non-fat milk dissolved in TBS containing 0.1% Tween-20 (TTBS) at 4 °C overnight and then incubated at room temperature with 0.5 g/mL anti-mouse iNOS antibodies (BD Biosciences, San Diego, CA, USA) for 2 h. The blots were washed and incubated with goat anti-mouse IgG conjugated to peroxidase in TTBS containing 5% non-fat milk for 1 h. Blots were washed in TTBS three times. The expression of each protein was detected by Amersham ECL prime western blotting detection reagent (GE Healthcare Life Sciences, Buckinghamshire, UK). 

#### 3.6.3. Carrageenan-Induced Hind Paw Edema in Rats

Anti-inflammatory activity of acetone extract from fruiting bodies of *P. florida* was assessed by carrageenan induced hind paw edema of rat model [36]. Rats were divided into five groups (five animals in each group) and acetone extracts (5, 15, and 50 mg/kg body weight, i.p.) were administered 30 min before the rats received 0.1 mL of carrageenan (1%, *w/v*) into the subplantar area of the right hind paw. A control group received the vehicle only and a positive control group was treated with indomethacin (5 mg/kg body weight, i.p.). The paw volume of rats was measured before injecting carrageenan (time 0) and at 2, 4, and 6 h after injection using a plethysmometer (MK-101P, Tokyo, Japan). The edema was expressed as the increase in paw volume, and the percentage of inhibition of edema was expressed as the reduction in volume with respect to the control group [31].

### 3.7. Statistical Analysis

Data are expressed as means ± standard deviations of three replicate determinations and were analyzed by SPSS V.13 (SPSS Inc., Chicago, IL, USA). One way Analysis of Variance (ANOVA) and Duncan’s new multiple range test were used to determine the differences among the means. Results were considered significant if *p*-values ≤ 0.05.

## 4. Conclusions

In conclusion, the antioxidant and anti-inflammatory activities of extracts from *P. florida* fruiting bodies were studied, and 13 phenolic compounds were detected. The antioxidant and anti-inflammatory activities of the extracts were supported by inhibition of iNOS expression and carrageenan-induced edema of the hind paw of rats. The result suggests that *P. florida* fruiting bodies are a good source of natural antioxidants and anti-inflammatory agents.
